# Cremation or Burial?

**Published:** 1892-12-03

**Authors:** 


### The Hospital

An Institutional Journal of

Science, Medicine, Nursing, and Philanthropy.

*For Hospitals, Asylums, Infirmaries, Dispensaries, Medical Practitioners, Students, Nurses, and the Charitable Public.*

Vol. XIII.—No. 323.] [Dec. 3, 1892.

### Cremation or Burial?

England is a small country with a large population; the disposal of the dead becomes, therefore, one of the most important of practical questions for the living. Are there any special principles which are of paramount weight in the consideration of the question? Ought we to be guided by sentiment or by pure reason, if there be such a thing in practice, or by custom and experience? The question has now attained the "burning" stage, if we may take Mr. Seymour Haden's fiery zeal, as displayed at the Society of Arts last week, to be the normal standard of feeling maintained by the opposed parties. Unfortunately—or was it fortunately?—Sir Henry Thompson was not present. If he had been the discussion would certainly have lacked nothing in point of warmth.

In the disposal of the dead the factor of sentiment ought to be considered, and that of national and religious custom as well as the scientific aspect of the case. Mr. Seymour Haden, who is an accomplished surgeon, stands for burial; but burial with a difference. Sir Henry Thompson, who is also an eminent surgeon, stands for cremation. It is extremely fortunate that both sides are represented by competent members of the medical profession. The subject is one which ought to enlist the keenest intelligence of medical men; and which, perhaps, they alone can bring to a scientific and generally approved conclusion.

It has been assumed as a matter of course that all the science is on the side of the cremationists, and all the sentiment on that of the burialists. Nobody who listened to Mr. Seymour Haden last week could persist in that delusion. At the first blush no doubt cremation has the appearance of being the more scientific method of disposing of dead bodies. That which is burnt appears to be destroyed, and although we know that it has not undergone, and cannot undergo actual destruction, yet we know also that its capacity for putrefaction has been entirely destroyed. When an organized body is thoroughly burnt there is nothing left behind of a putrefactive nature. Nevertheless, there is still to be considered the important question of the products of combustion, the smoke and other constituents which are belched forth into the air. Of course, at the present time those products are insignificant in their consequences, because they are insignificant in their quantity. We do not cremate in all Great Britain more than a few scores, or, at most, hundreds of persons in a whole year. But what sort of an atmosphere should we create if hundreds of tall chimneys were vomiting forth the smoke products of thousands of dead human bodies every day, and all the year round?

On the other hand, the placing of dead bodies in the ground appears to be a method which must result in poisoning, not only the soil, but any water which may be near, and not only the water, but the atmosphere above the ground by the escape of gases. But is this actually the case? Mr. Seymour Haden says emphatically that it is not, and in support of his contention he adduces two classes of reasons which cannot but be of weight with practical and scientific people. To set forth the matter briefly: when a human or other animal body is put naked into the ground it rapidly decomposes: some of its constituents are taken up by the surrounding soil, others pass through the soil, as gases, into the air. Now, the questions are, is the soil, as soil, injured by what it takes up from the decaying body; and is the air, as air, injured by the gases which rise through the soil and mix with it? Mr. Seymour Haden says "No!" We should be inclined to say "No!" and "Yes!" The proper answer would seem to be that it depends upon the number of bodies buried in a given quantity of soil, and upon the mode of burial. Put a reasonable number of bodies into the soil, enclose them in rapidly perishable cases, as Mr. Seymour Haden suggests, and it will be found, as a matter of fact, that they do not injure either soil, or air, or neighbouring water. On the contrary, the soil, at any rate, will be decidedly improved.

The other class of reasons insisted upon by Mr. Seymour Haden consists of the facts of common experience. The world, that is, the historical world, is now at least six thousand years old. Has it ever been known that burial grounds, under reasonably sanitary conditions, have done practical harm to soil, water, or air? No doubt under foolish conditions harm may have been done to all these. But such conditions, though prevalent enough in the past, are by no means likely to be prevalent in the future. Mr. Haden goes perhaps further than the facts warrant when he tells us that even the thousands slain in battle, when heaped up and buried in the most hurried and unscientific way, have practically never done any great amount of harm to those living in the vicinity. We should be inclined to doubt that; though when we come to look for actual facts which prove that real harm has been done to the living by such indiscriminate burials of large numbers of the dead, it is astonishing how very few facts which have been scientifically verified are available for the purposes of the discussion.

"Cremation is an incentive to crime." That was the title and the leading contention of Mr. Haden's paper. Such a contention cannot for a moment be seriously doubted. The partisans of cremation may minimise the facts, but they cannot deny their reality. It is open to anybody also to contend that the discovery or non-discovery of a few murderers is as nothing compared with the importance of pure air and wholesome drinking water. But here again it is easy for the hasty to be misled. The cremationists contend that very few bodies are exhumed after death, and that very few murderers are convicted who would escape if cremation were universal. But Mr. Seymour Haden is most energetic and even denunciatory on the other side. According to him, the statistics of the cremationists are quite untrustworthy; and in cases where they would assert that only one murder had been brought to light by the exhumation of the dead, he would claim at least ten. And, with regard to pure air, he contends that cremation carried out on the same scale as interment, would poison the air, not less, but more than burial.

If we allow ourselves to be blinded by the dust of partisanship we shall come to the conclusion that the subject is one of extreme practical difficulty. "Who shall decide when doctors disagree?" But doctors are, in fact, disagreeing every day; and yet "issues of great pith and moment" are constantly being decided without any injurious consequences whatsoever. The arguments of the cremationists and the burialists, when balanced against each other, produce this kind of nett general impression in the non-contentious mind—that it is not a matter of any urgency whether we cremate the dead or not, but that it is a matter of very great urgency that we carry out sanitary burial. Sanitary burial is the kind of burial which Mr. Seymour Haden insists upon. In the first place he contends that we should bury the dead as soon as possible after it is certain that they are really dead; that is to say, as soon as they have passed through the stage of *rigor mortis.* Almost more important than this is the coffin in which the dead are to be buried. Not only should the coffin not be of lead, or of oak, or other hard material, but it should not be of a durable substance at all. The one rule about coffins is that they be as perishable as possible. The bodies of the dead should come into contact with "mother earth" at the earliest possible moment. "Earth to earth" is the standard of sanitary perfection in burials. The soil selected for burial grounds should be dry and porous. Burial grounds may be filled, but should not be overcrowded. Let but these conditions be intelligently carried out, and the dead may continue to be buried, rather than burnt, to the end of time. In very crowded places where burying ground is difficult to obtain, cremation may constitute a useful adjunct; but in ordinary circumstances, where burial can be sanitarily conducted, the bodies of our dead may continue to be "committed to the ground," there to undergo the slow resolution into their elements, which we all feel to be in keeping alike with their wishes and our own.

